# Supplementary material for: Mechanistic insights into the structure-based design of a CspZ-targeting Lyme disease vaccine
Source: Nat Commun. 2025 Apr 7;16:2898. doi: 10.1038/s41467-025-58182-x (PMC11973211; doi:10.1038/s41467-025-58182-x)
Supplement: Supplementary file 1 — Supplementary Information [file 41467_2025_58182_MOESM1_ESM.pdf]

Supplementary Information

Supplementary figures and figure legends

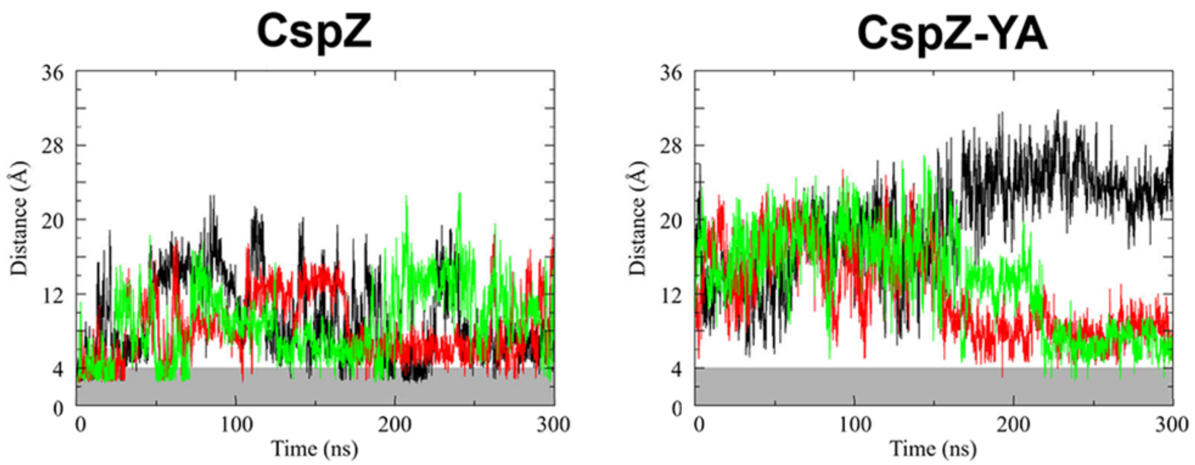

Figure S1. MD simulations of CspZ and CspZ-YA suggest the salt bridge between E186 and R206 is unlikely to be formed in CspZ-YA. MD simulations of **(A)** CspZ and **(B)** CspZ-YA, showed the distance between the side chains of residues E186 and R206 over 300 ns. Proximity below 4 Å, corresponding to salt bridge is shaded in grey. Different colors (black, green, red) represent three independent runs.

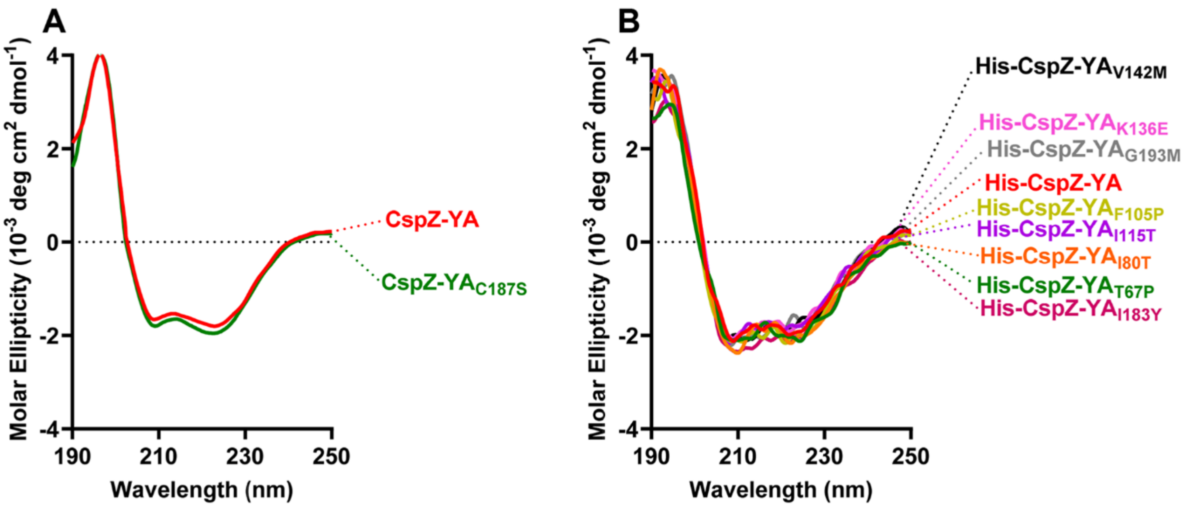

Figure S2. CD spectra demonstrate no impacts of secondary structures by mutating indicating amino acids of CspZ-YA. Far-UV CD analysis of **(A)** untagged CspZ-YA and CspZ-YA$_{C187S}$ (CspZ-YA$_{C187S}$), and **(B)** histidine tagged CspZ-YA (His-CspZ-YA) and the mutant proteins derived from this protein. The molar ellipticity, Φ, was measured from 190-250nm for 10μM of each protein in PBS. Source data are provided as a Source Data file.

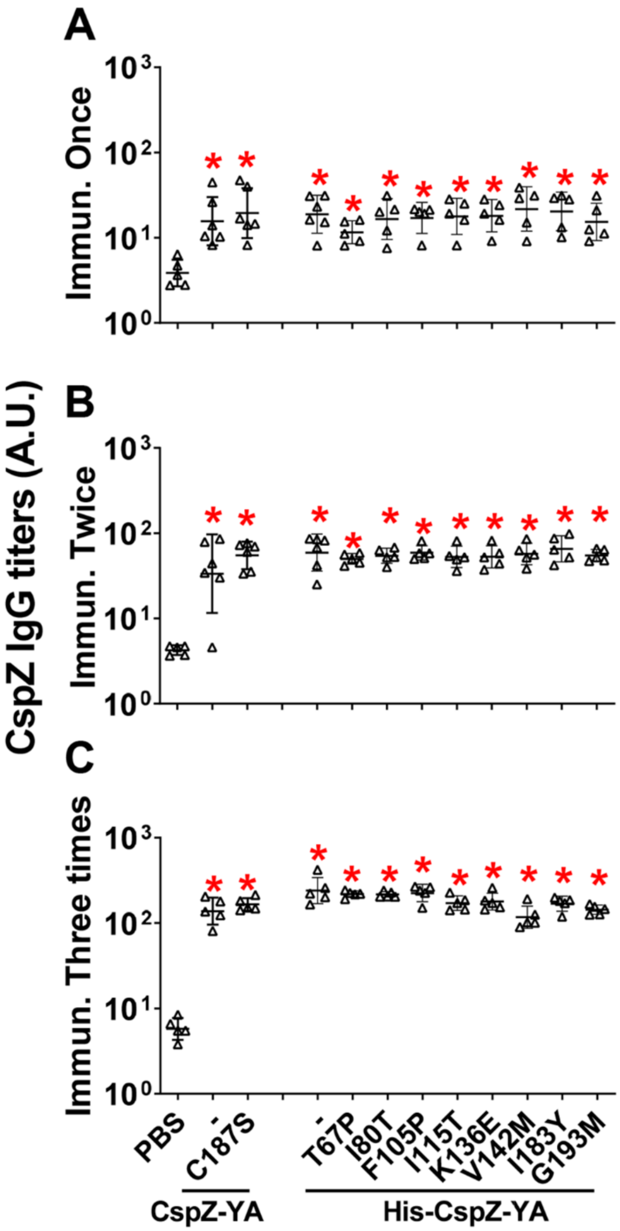

Figure S3. Immunization of CspZ-YA and its mutant proteins triggered indistinguishable levels of antibodies against CspZ. Sera were collected at 14dpli from pre-adolescent C3H/HeN mice immunized **(A)** once, **(B)** twice, or **(C)** three times in the fashion as described in Fig. 1. These mice were immunized with PBS (control) or untagged CspZ-YA or its derived mutant protein, or histidine tagged CspZ-YA (His-CspZ-YA), or its derived mutant proteins (Six mice for CspZ-YA- or CspZ-YA$_{C187S}$-immunized mice whereas five mice for the rest of immunization groups of mice).

The levels of total IgG against CspZ were determined using quantitative ELISA. Data shown are the geometric mean ± geometric standard deviation of the titers of anti-CspZ antibodies from n = 6 mice immunized once or twice or n = 5 mice immunized three times with CspZ-YA, CspZ-YA_{C187S} or His-CspZ-YA or n = 5 mice inoculated with each of other proteins or PBS. Asterisks indicate the statistical significances ($p < 0.05$, Kruskal Wallis test with the two-stage step-up method of Benjamini, Krieger, and Yekutieli) of differences in antibody titers relative to the sera from PBS-inoculated mice. **(A)** PBS: CspZ-YA, C187S, His-CspZ-YA, T67P, I80T, F105P, I115T, K136E, V142M, I183Y, G193M p = 0.013, 0.002, 0.0017, 0.0129, 0.0092, 0.0071, 0.0049, 0.0044, 0.0007, 0.0014, 0.0143. **(B)** PBS: CspZ-YA, C187S, His-CspZ-YA, T67P, I80T, F105P, I115T, K136E, V142M, I183Y, G193M p = 0.0466, 0.0036, 0.0006, 0.0361, 0.0085, 0.0036, 0.0169, 0.0154, 0.0045, 0.0004, 0.0009. **(C)** PBS: CspZ-YA, C187S, His-CspZ-YA, T67P, I80T, F105P, I115T, K136E, V142M, I183Y, G193M p = 0.0361, 0.0272, < 0.0001, < 0.0001, < 0.0001, < 0.0001, 0.0134, 0.0076, 0.0429, 0.0177, 0.0411. Source data are provided as a Source Data file.

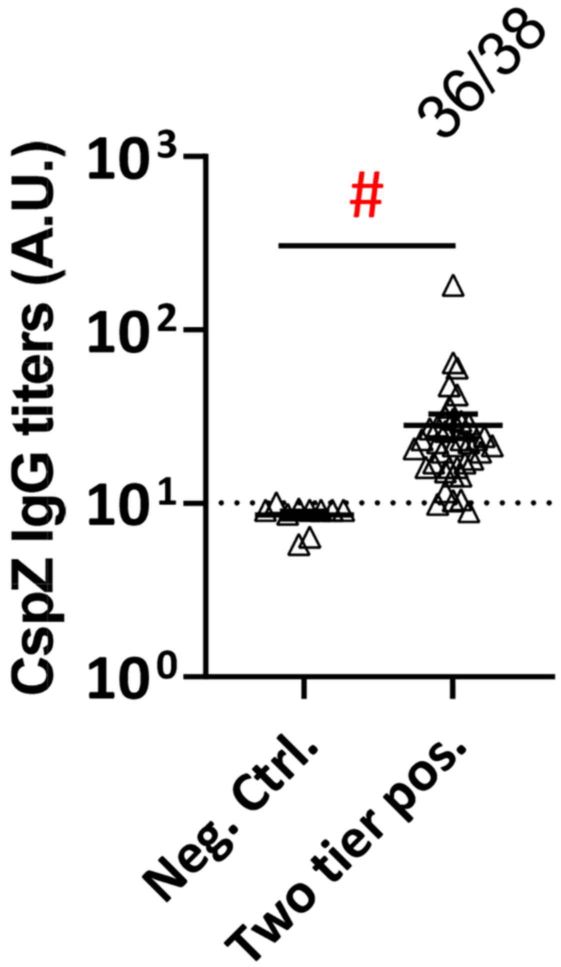

Figure S4. More than 90% of Lyme disease human patients develop CspZ antibodies. Sera from patients with seropositive for Lyme disease infection (Two tier pos.; Positive in Two tier test) were determined for the titers of antibodies that recognize untagged CspZ using ELISA, as described in the section "ELISA" of the Materials and Methods. The serum samples from humans residing in non-endemic area of Lyme disease were included as negative control (Neg. ctrl.) and to set up the threshold value of titers that can be used to determine CspZ antibody positivity. That threshold value was mean 1.5-folds of standard deviation extrapolated from the values of negative control human sera. Thirty six out of 38 serum samples (94.7%) yield greater anti-CspZ IgG titers

than the threshold values and was thus considered positive for CspZ antibodies. Shown is the geometric mean ± geometric standard deviation of the titers from sera of 38 patients or 10 negative control humans. Statistical significance ($p < 0.05$, Mann-Whitney test) of differences in CspZ-IgG titers between groups are indicated ("#"). Neg. Ctrl.: Two tier pos. $p < 0.0001$ Source data are provided as a Source Data file.

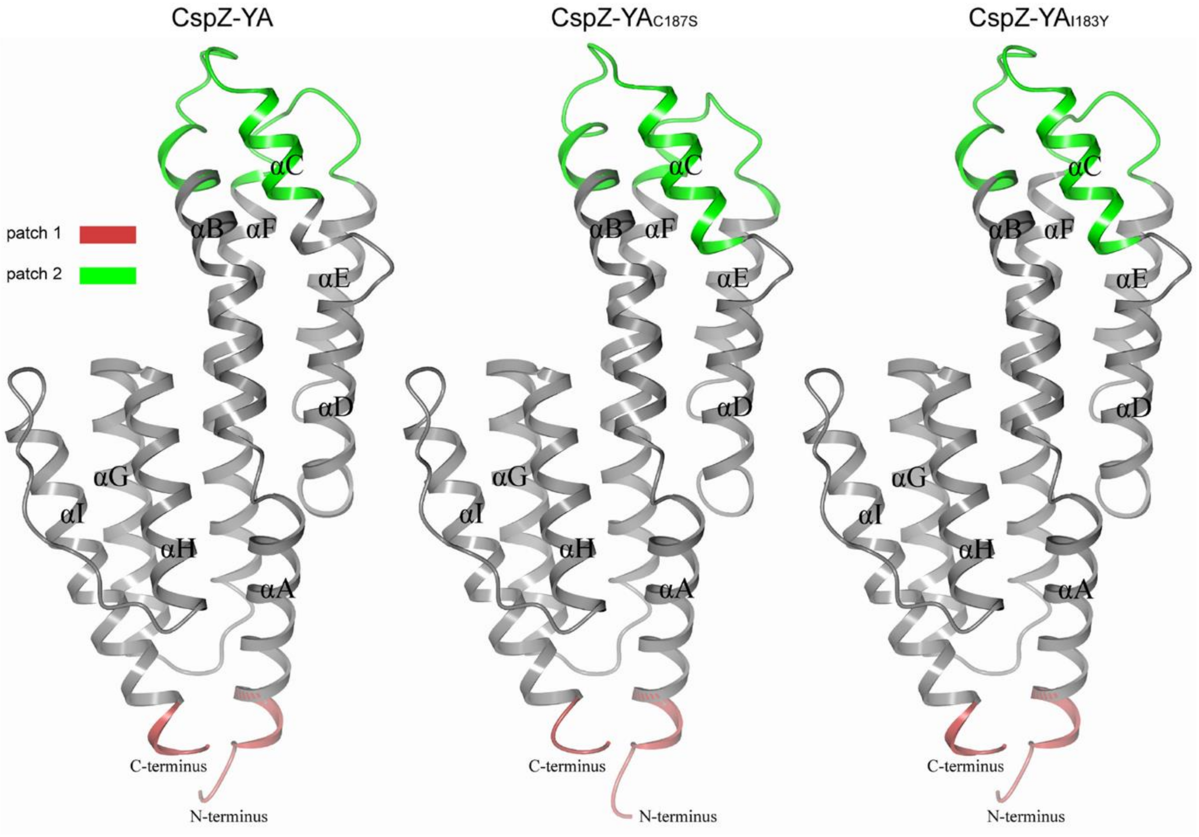

Figure S5. The epitopes of CspZ-YA, CspZ-YA$_{C187S}$, and CspZ-YA$_{I183Y}$ were predicted based on MLCE. The crystal structures of CspZ-YA (PDB ID 9F1V), CspZ-YA$_{C187S}$ (PDB ID 9F21) and the predicted structure of CspZ-YA$_{I183Y}$ were mapped for the putative immunogenic epitope patches as predicted by the matrix of lowest coupling energies (MLCE). All nine α-helices of CspZ variants are labeled (αA-αI).

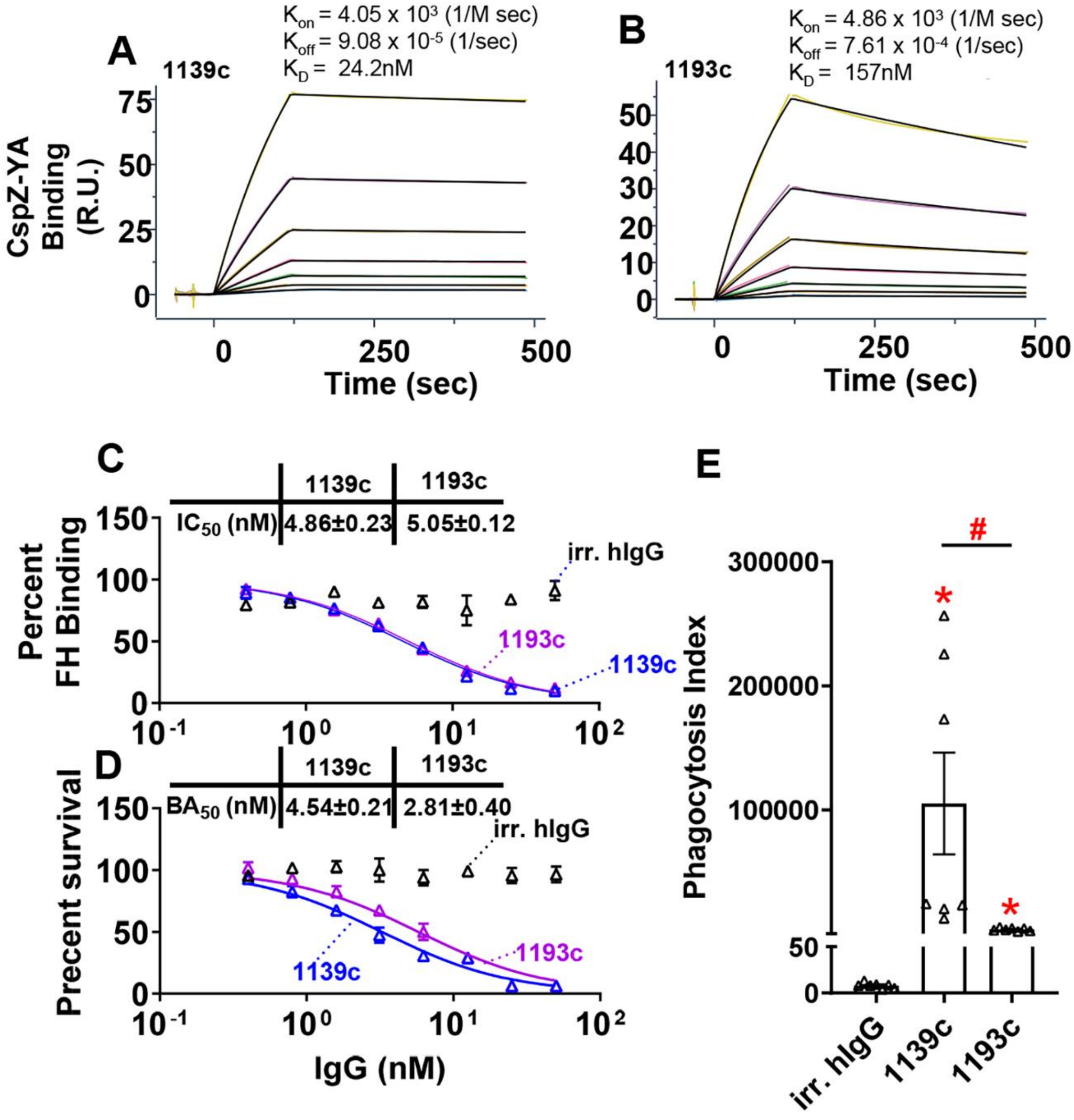

Figure S6. The humanized chimeric monoclonal antibodies 1139c and 1193c efficiently recognize CspZ-YA, prevent human FH-binding, and promote lysis and opsonophagocytosis of *B. burgdorferi*. (A and B) The humanized chimeric monoclonal antibody **(A)** #1139c or **(B)** #1193c was flowed over the chip surface, conjugated with indicated untagged CspZ-YA. Binding was measured in response units (R.U.) by surface plasmon resonance. Shown is the mean ± standard deviation of the k_{on}, k_{off}, and K_D values extrapolated from n = 3 experiments. One

represented experiment is shown in this panel. **(C)** The monoclonal antibody #1139c or #1193c, or irrelevant human IgG (control, irr. hIgG) at indicated concentrations or PBS (control, data not shown) was added into the CspZ-coated ELISA plate wells. Each of those wells was then incubated with human FH, and the levels of bound FH were quantified using sheep anti-human FH and goat anti-sheep HRP IgG as primary and secondary antibodies, respectively. The work was performed on n = 3 independent experiments; within each experiment, samples were run in triplicate. Data are expressed as the percent human FH binding, derived by normalizing the levels of bound human FH from IgG-treated wells to that from PBS-treated wells. Data shown are the mean ± SEM of the percent human FH binding from n = 3 replicates. Shown is one representative experiment. The concentrations of the IgG to inhibit 50% of human FH bound by CspZ (IC_{50}) was obtained from curve-fitting and shown in the inlet figure. The IC_{50} values are shown as the mean ± SD of from three experiments. **(D)** The monoclonal antibody #1139c or #1193c, or irrelevant human IgG (control, irr. hIgG) or PBS (control, data not shown) were serially diluted as indicated, and mixed with guinea pig complement and *B. burgdorferi* strains B31-A3 (5×10^5 cells ml^{-1}). After incubated for 24 hours, surviving spirochetes were quantified from three fields of view for each sample using dark-field microscopy. The work was performed on three independent experiments. The survival percentage was derived from the proportion of IgG-treated to PBS-treated spirochetes. Shown is one representative experiment, and in that experiment, the data points are the mean ± SEM of the survival percentage from three replicates. The 50% borreliacidal activity of each IgGs (BA_{50}), representing the IgG concentrations that effectively killed 50% of spirochetes, was obtained and extrapolated from curve-fitting and shown in the inlet figure. The BA_{50} values are shown as the mean ± SD of from n = 3 experiments. **(E)** Human sera in the presence of irrelevant human IgG (control), 1139c, or 1193c (2μM) were incubated with PMNs (5×10^6 cells ml^{-1}) and

indicated CFSE-labeled *B. burgdorferi* B31-A3 (5×10^7 cells ml^{-1}). The resulting *B. burgdorferi*-PMN mixtures were applied to flow cytometry after 10 min of incubation at 37°C to obtain the mean fluorescence intensity (MFI) values. The MFI values derived from *B. burgdorferi*-PMN mixtures incubated at 4°C throughout the experiment are included as control. The MFI values of each of the *B. burgdorferi*-PMN mixtures in 37°C and 4°C were used to calculate "Phagocytic index" as described in "Materials and Methods" to quantitatively show the levels of phagocytosis. This assay was performed in four independent determinations, and shown is one representative experiment. The panel represents the mean of phagocytic index ± SEM from n = 7 independent determinations. Asterisks indicate the statistical significance ($p < 0.05$, Kruskal Wallis test with the two-stage step-up method of Benjamini, Krieger, and Yekutieli) of differences in phagocytic index relative to irrelevant human IgG-treated Lyme borreliae. **(E)** irr. hIgG: 1139c, 1193c p = < 0.0001, 0.0348. 1139c: 1193c p = 0.0348. Source data are provided as a Source Data file.

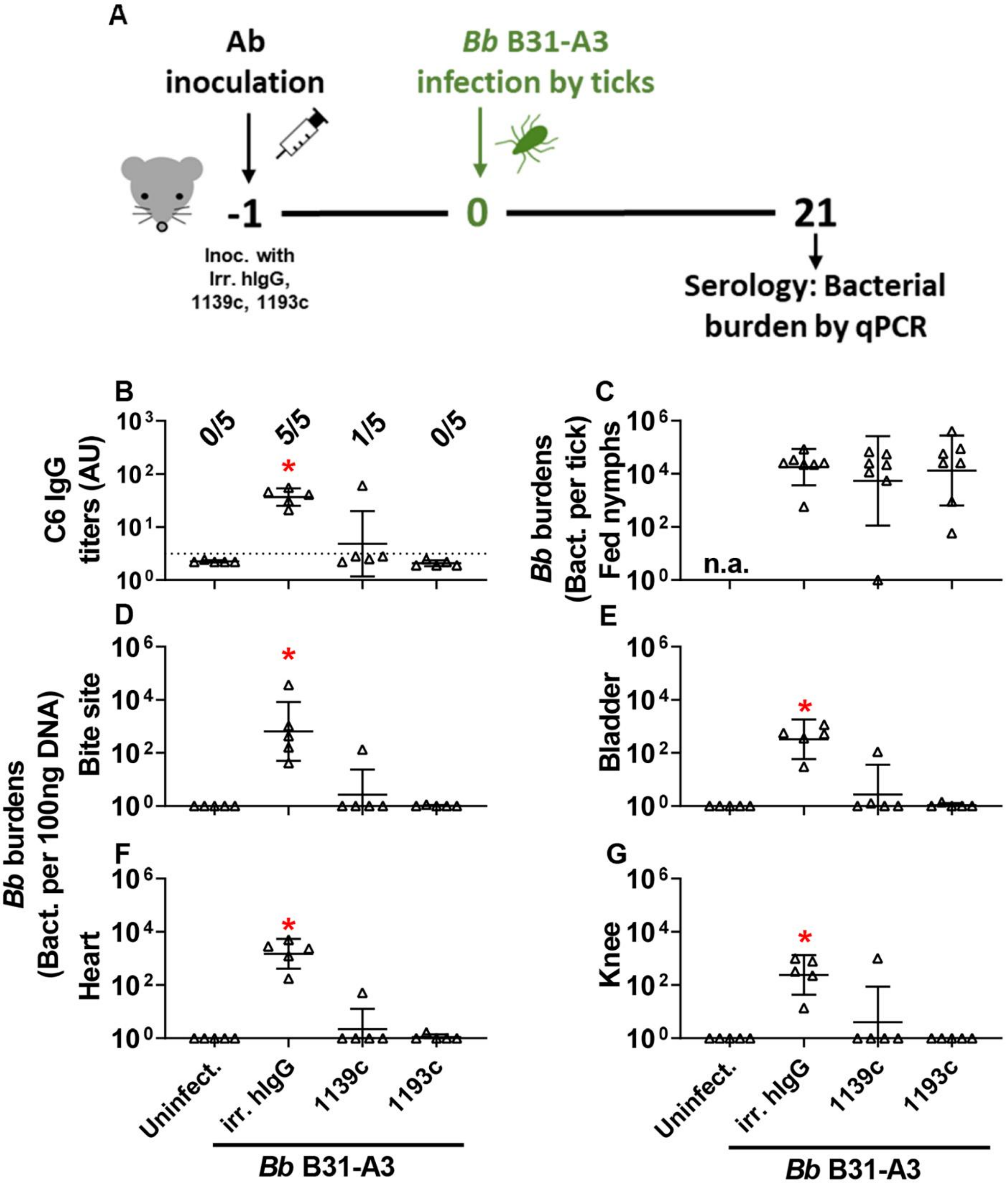

Figure S7. The humanized chimeric monoclonal antibodies 1139c and 1193c prevent seroconversion and tissue colonization caused by *B. burgdorferi* B31-A3 infection. (A) Timeframe of the IgG inoculation and *B. burgdorferi* infection. **(B to G)** Pre-adolescent C3H/HeN

mice were inoculated with the monoclonal antibody #1139c or #1193c, or irrelevant human IgG (control, irr. hIgG) at the dose of 1 mg/kg. At 24 hours after IgG inoculation, these mice were fed on by *I. scapularis* nymphs carrying *B. burgdorferi* B31-A3 (*Bb* B31-A3). An additional mice inoculated with PBS but not fed on by ticks were included as the control (Uninfect.). The tissues were collected from those mice at 4 days post nymph feeding. Spirochete burdens at **(B)** the tick feeding site ("Bite Site"), **(C)** bladder, **(D)** heart, and **(E)** knees were quantitatively measured at 21 dpf, shown as the number of spirochetes per 100ng total DNA. Data shown are the geometric mean ± geometric standard deviation of the spirochete burdens from n = 7 nymphs per group or n = 5 mice per group. Statistical significances ($p < 0.05$, Kruskal-Wallis test with the two-stage step-up method of Benjamini, Krieger, and Yekutieli) of differences in bacterial burdens relative to (*) uninfected mice are presented. **(B)** Uninfect.: irr. hIgG p = 0.0078. **(D)** Uninfect.: irr. hIgG p = 0.0007. **(E)** Uninfect.: irr. hIgG p = 0.0007. **(F)** Uninfect.: irr. hIgG p = 0.0006. **(G)** Uninfect.: irr. hIgG p = 0.0017. Source data are provided as a Source Data file.

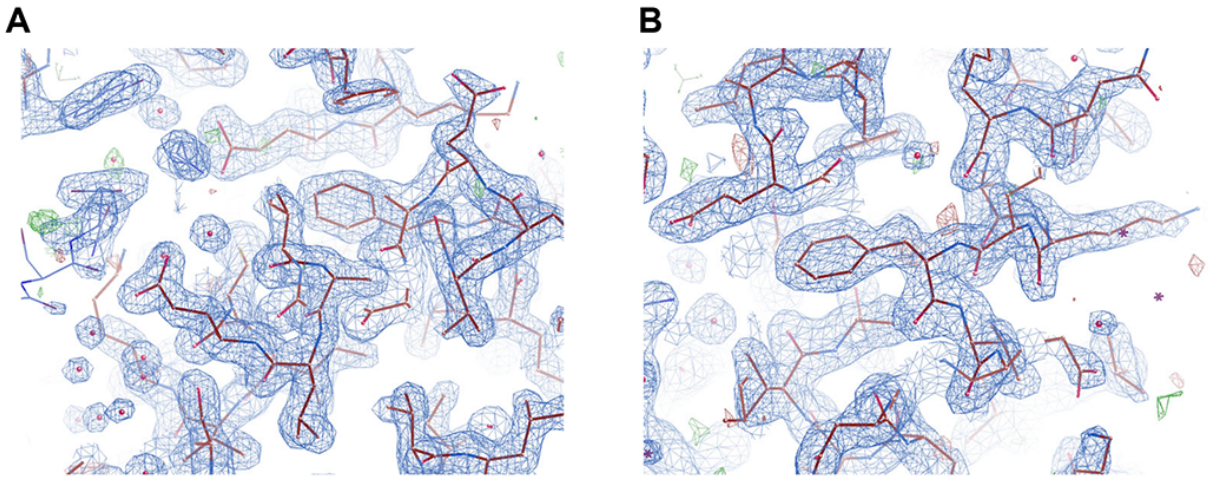

Figure S8. The electron density maps of the portions of the crystal structures of CspZ-YA and CspZ-YA$_{C187S}$. The electron density maps of newly resolved crystal structures of CspZ-YA proteins in this study. Shown are the portions of the crystal structures of **(A)** CspZ-YA and **(B)** CspZ-YA$_{C187S}$.

Supplementary Tables

Table S1. Data processing, refinement, and validation statistics for the crystal structures.

**Dataset**	**CspZ-YA**	**CspZ-YA$_{C187S}$**
**X-ray diffraction data**		
PDB entry	9F1V	9F21
Beamline	Diamond Light Source beamline I03	BESSY II beamline 14.1
Space group	$P2_12_12_1$	$P2_12_12_1$
a, *b*, *c* (Å)	31.47, 41.55, 162.56	31.55, 41.68, 162.81
Matthews coefficient	2.32	2.15
Solvent content in the crystal (%)	47.0	42.8
Wavelength (Å)	0.9762	0.9184
Resolution (Å)	162.56-1.90	41.68-1.95
Highest resolution bin (Å)	1.94-1.90	2.00-1.95
No. of reflections	231969 (15040)	205571 (15186)
No. of unique reflections	17550 (1116)	16483 (1144)
Completeness (%)	99.5 (100.0)	99.9 (100.0)
R_{merge}[a]	0.09 (0.46)	0.09 (0.69)
$CC_{1/2}$[b]	0.998 (0.972)	0.998 (0.971)
$I/\sigma (I)$	15.5 (4.8)	18.1 (3.6)
Multiplicity	13.2 (13.5)	12.5 (13.3)
**Refinement**		
R_{work}	0.187 (0.245)	0.230 (0.318)
R_{free}^{Δ}[c]	0.241 (0.316)	0.255 (0.376)
**Average B-factor (Å^2)**		

Overall	28.0	30.0
From Wilson plot	17.0	17.5
No. of atoms		
Protein	1743	1743
Water	198	179
**RMS deviations from ideal**		
Bond lengths (Å)	0.009	0.009
Bond angles (^{o})	1.509	1.580
**Ramachandran outliers (%)**		
Residues in most favored regions (%)	97.67	95.35
Residues in allowed regions (%)	2.33	4.65
Outliers (%)	0	0

[a]$R_{merge} = \Sigma_{hkl} \Sigma_i |I_i(hkl) - <I(hkl)>| / \Sigma_{hkl} \Sigma_i |I_i(hkl)|$ were $|I_i(hkl)|$ is the observed intensity and $<I(hkl)>$ is the mean intensity [1].

[b]$CC_{1/2} = \Sigma_{hkl} [(I_A - <I_A>)(I_B - <I_B>)] / \Sigma_{hkl} (I_A - <I_A>)^2 \Sigma_{hkl} (I_B - <I_B>)^2$ were I_A and I_B are the mean intensities of the two half-datasets for reflection *hkl*, and $<I_A>$ and $<I_B>$ are the average intensities for each half-dataset [2].

[c]For the calculation of R_{free}, 5% of the relflections were randomly selected and excluded from the refinement process. Values in parentheses are for the highest resolution bin. $^{\Delta}R_{free} = \Sigma_{test\ set} |F_{obs}(hkl) - F_{calc}(hkl)| / \Sigma_{test\ set} F_{obs}(hkl)$ where F_{obs} are the observed structure factor amplitudes, and F_{calc} are the calculated apmlitudes from the model [3].

Table S2. BA_{50} values of the sera derived from mice immunized with CspZ-YA proteins in different immunization frequency

BA_{50}[a]	CspZ-YA[b]		His-CspZ-YA[c]								
	**-**	**C187S**	**-**	**T67P**	**I80T**	**F105P**	**I115T**	**K136E**	**V142M**	**I183Y**	**G193M**
**Immunization frequency** 1[d]	25±1.6	19±1.2	26±1.1	20±1.5	24±1.6	29±1.0	25±1.2	17±1.2	21±1.3	29±1.3	27±1.1
2[d]	43±1.3	204±1	43±1.5	45±1.1	56±1.1	53±1.0	53±1.3	41±1.1	35±1.3	164±1.0	48±1.0
3[d]	232±1.3	832±1	259±1	241±1.2	202±1.3	268±1.6	287±1.3	223±1.4	239±1.4	1306±1.4	238±1.5

[a]The dilution rate of the sera that kill 50% of *B. burgdorferi* B31-A3. Shown is the mean ± standard deviation of the BA_{50} values derived from three experiments (three replicates per experiment).

[b]Sera from the mice immunized with Untagged CspZ-YA

[c]Sera from the mice immunized with histidine-tagged CspZ-YA

[d]1, 2, and 3 indicate the mice immunized with indicated antigen once, twice, and three times, respectively, as described in Fig. 1.

Table S3. The thermostability of CspZ-YA proteins.

	CspZ-YA		His-CspZ-YA	
	-	C187S	-	I183Y
**Tm (ºC)**[a]	58.4±0.2	62.7±0.1	57.5±1.1	61.8±0.1

[a]Shown is the mean ± standard deviation of the Tm values derived from six experiments (one replicate per experiment).

[b]Histidine-tagged CspZ-YA

Table S4. Strains and plasmids used in this study.

Strain or plasmid	Genotype or characteristic	Source
<u>*B. burgdorferi*</u>		
B31-A3	Clone A3 of *B. burgdorferi* B31 isolated from *I. scapularis* ticks in US.	[4]
<u>*E. coli*</u>		
BL21(DE3)	F−, *ompT hsdSB* (rB− mB−) *gal dcm* (DE3)	EMD Millipore
BL21(DE3)/pET28a-CspZ	BL21(DE3) producing residues 19 to 237 of CspZ from *B. burgdorferi* B31-A3 (GenBank# AAC65998.1)	[5]
BL21(DE3)/pET28a-CspZ-YA	BL21(DE3) producing residues 19 to 237 of CspZ (GenBank# AAC65998.1) with tyrosine-207 and -211 simultaneously replaced by alanine residues (CspZ-YA)	[5]
BL21(DE3)/pET41a-CspZ-YA	BL21(DE3) producing residues 19 to 237 of CspZ (GenBank# AAC65998.1) with tyrosine-207 and -211 simultaneously replaced by alanine residues (CspZ-YA)	[6]
BL21(DE3)/pET41a-CspZ-YA$_{C53S}$	BL21(DE3) producing residues 19 to 237 of CspZ-YA with cysteine-53 replaced by serine	This study
BL21(DE3)/pET28a-CspZ-YA$_{T67P}$	BL21(DE3) producing residues 19 to 237 of CspZ-YA with threonine-67 replaced by proline	This study
BL21(DE3)/pET28a-CspZ-YA$_{I80T}$	BL21(DE3) producing residues 19 to 237 of CspZ-YA with isoleucine-80 replaced by threonine	This study
BL21(DE3)/pET28a-CspZ-YA$_{F105P}$	BL21(DE3) producing residues 19 to 237 of CspZ-YA with phenylalanine-105 replaced by proline	This study
BL21(DE3)/pET28a-CspZ-YA$_{I115T}$	BL21(DE3) producing residues 19 to 237 of CspZ-YA with isoleucine-115 replaced by threonine	This study
BL21(DE3)/pET28a-CspZ-YA$_{K136E}$	BL21(DE3) producing residues 19 to 237 of CspZ-YA with lysine-136 replaced by glutamate	This study
BL21(DE3)/pET28a-CspZ-YA$_{V142M}$	BL21(DE3) producing residues 19 to 237 of CspZ-YA with valine-142 replaced by methionine	This study

BL21(DE3)/pET28a-CspZ-YA$_{I183Y}$	BL21(DE3) producing residues 19 to 237 of CspZ-YA with isoleucine-183 replaced by tyrosine	This study
BL21(DE3)/pET41a-CspZ-YA$_{C187S}$	BL21(DE3) producing residues 19 to 237 of CspZ-YA with cysteine-187 replaced by tyrosine	This study
BL21(DE3)/pET28a-CspZ-YA$_{G139M}$	BL21(DE3) producing residues 19 to 237 of CspZ-YA with glycine-139 replaced by methionine	This study
<u>Plasmids</u>		
pET28a-CspZ	KanR[a]; pET28a encoding protein residue 19 to 237 of CspZ from *B. burgdorferi* B31-A3 (GenBank# AAC65998.1)	[5]
pET28a-CspZ-YA	KanR; pET28a encoding protein residue 19 to 237 of CspZ (GenBank# AAC65998.1) with tyrosine-207 and -211 simultaneously replaced by alanine residues (CspZ-YA)	[5]
pET41a-CspZ-YA	KanR; pET41a encoding protein residue 19 to 237 of CspZ (GenBank# AAC65998.1) with tyrosine-207 and -211 simultaneously replaced by alanine residues (CspZ-YA)	[6]
pET41a-CspZ-YA$_{C53S}$	KanR; pET41a encoding protein residue 19 to 237 of CspZ-YA with cystein-53 replaced by serine	This study
pET28a-CspZ-YA$_{T67P}$	KanR; pET28a encoding protein residue 19 to 237 of CspZ-YA with threonine-67 replaced by proline	This study
pET28a-CspZ-YA$_{I80T}$	KanR; pET28a encoding protein residue 19 to 237 of CspZ-YA with isoleucine-80 replaced by threonine	This study
pET28a-CspZ-YA$_{F105P}$	KanR; pET28a encoding protein residue 19 to 237 of CspZ-YA with phenylalanine-105 replaced by proline	This study
pET28a-CspZ-YA$_{I115T}$	KanR; pET28a encoding protein residue 19 to 237 of CspZ-YA with isoleucine-115 replaced by threonine	This study
pET28a-CspZ-YA$_{K136E}$	KanR; pET28a encoding protein residue 19 to 237 of CspZ-YA with lysine-136 replaced by glutamate	This study
pET28a-CspZ-YA$_{V142M}$	KanR; pET28a encoding protein residue 19 to 237 of CspZ-YA with valine-142 replaced by methionine	This study

pET28a-CspZ-YA_{I183Y}	KanR; pET28a encoding protein residue 19 to 237 of CspZ-YA with isoleucine-183 replaced by tyrosine	This study
pET41a-CspZ-YA_{C187S}	KanR; pET41a encoding protein residue 19 to 237 of CspZ-YA with cysteine-187 replaced by serine	This study
pET28a-CspZ-YA_{G139M}	KanR; pET28a encoding protein residue 19 to 237 of CspZ-YA with glycine-139 replaced by methionine	This study

[a] Kanamycin resistant

Reference

1. Evans P. Scaling and assessment of data quality. *Acta crystallographica Section D, Biological crystallography* **62**, 72-82 (2006).

2. Karplus PA, Diederichs K. Linking crystallographic model and data quality. *Science* **336**, 1030-1033 (2012).

3. Brunger AT. Free R value: a novel statistical quantity for assessing the accuracy of crystal structures. *Nature* **355**, 472-475 (1992).

4. Elias AF*, et al.* Clonal polymorphism of Borrelia burgdorferi strain B31 MI: implications for mutagenesis in an infectious strain background. *Infection and immunity* **70**, 2139-2150 (2002).

5. Marcinkiewicz AL*, et al.* Blood treatment of Lyme borreliae demonstrates the mechanism of CspZ-mediated complement evasion to promote systemic infection in vertebrate hosts. *Cellular microbiology* **21**, e12998 (2019).

6. Chen YL, *et al.* Biophysical and biochemical characterization of a recombinant Lyme disease vaccine antigen, CspZ-YA. *International journal of biological macromolecules* **259**, 129295 (2024).
