# Supplementary material for: Mechanistic insights into the structure-based design of a CspZ-targeting Lyme disease vaccine
Source: Nat Commun. 2025 Apr 7;16:2898. doi: 10.1038/s41467-025-58182-x (PMC11973211; doi:10.1038/s41467-025-58182-x)
Supplement: Supplementary file 3 — Description of Additional Supplementary Files [file 41467_2025_58182_MOESM3_ESM.pdf]

Description of Additional Supplementary Files

File Name: Supplementary Movie 1
Description: Molecular dynamics simulations of CspZ performed for 300 nanoseconds at 300 K.

File Name: Supplementary Movie 2
Description: Molecular dynamics simulations of CspZ-YA performed for 300 nanoseconds at 300 K

File Name: Supplementary Movie 3
Description: Molecular dynamics simulations of CspZ-YAC187S performed for 300 nanoseconds at 300 K

File Name: Supplementary Movie 4
Description: Molecular dynamics simulations of CspZ-YAI183Y performed for 300 nanoseconds at 300 K.
