# Supplementary material for: Mechanistic insights into the structure-based design of a CspZ-targeting Lyme disease vaccine
Source: Nat Commun. 2025 Apr 7;16:2898. doi: 10.1038/s41467-025-58182-x (PMC11973211; doi:10.1038/s41467-025-58182-x)
Supplement: Supplementary file 8 — Reporting Summary [file 41467_2025_58182_MOESM8_ESM.pdf]

nature portfolio

Corresponding author(s): Kalvis Brangulis, Ching-Lin Hsieh, Wen-Hsiang Chen, Yi-Pin Lin

Last updated by author(s): Feb 23, 2025

Reporting Summary

Nature Portfolio wishes to improve the reproducibility of the work that we publish. This form provides structure for consistency and transparency in reporting. For further information on Nature Portfolio policies, see our Editorial Policies and the Editorial Policy Checklist.

Statistics

For all statistical analyses, confirm that the following items are present in the figure legend, table legend, main text, or Methods section.

n/a	Confirmed	
☐	☒	The exact sample size (*n*) for each experimental group/condition, given as a discrete number and unit of measurement
☐	☒	A statement on whether measurements were taken from distinct samples or whether the same sample was measured repeatedly
☐	☒	The statistical test(s) used AND whether they are one- or two-sided<br>*Only common tests should be described solely by name; describe more complex techniques in the Methods section.*
☒	☐	A description of all covariates tested
☐	☒	A description of any assumptions or corrections, such as tests of normality and adjustment for multiple comparisons
☐	☒	A full description of the statistical parameters including central tendency (e.g. means) or other basic estimates (e.g. regression coefficient) AND variation (e.g. standard deviation) or associated estimates of uncertainty (e.g. confidence intervals)
☐	☒	For null hypothesis testing, the test statistic (e.g. *F*, *t*, *r*) with confidence intervals, effect sizes, degrees of freedom and *P* value noted<br>*Give P values as exact values whenever suitable.*
☒	☐	For Bayesian analysis, information on the choice of priors and Markov chain Monte Carlo settings
☒	☐	For hierarchical and complex designs, identification of the appropriate level for tests and full reporting of outcomes
☐	☒	Estimates of effect sizes (e.g. Cohen's *d*, Pearson's *r*), indicating how they were calculated

Our web collection on statistics for biologists contains articles on many of the points above.

Software and code

Policy information about availability of computer code

Data collection	N/A
Data analysis	The statistical analysis in this work was performed using GraphPad Prism 9.3.1.

For manuscripts utilizing custom algorithms or software that are central to the research but not yet described in published literature, software must be made available to editors and reviewers. We strongly encourage code deposition in a community repository (e.g. GitHub). See the Nature Portfolio guidelines for submitting code & software for further information.

Data

Policy information about availability of data

All manuscripts must include a data availability statement. This statement should provide the following information, where applicable:

- Accession codes, unique identifiers, or web links for publicly available datasets
- A description of any restrictions on data availability
- For clinical datasets or third party data, please ensure that the statement adheres to our policy

The coordinates and the structure factors for CspZ-YA and CspZ-YAC187S have been deposited in the Protein Data Bank with accession codes 9F1V and 9F21, respectively.

Research involving human participants, their data, or biological material

Policy information about studies with human participants or human data. See also policy information about sex, gender (identity/presentation), and sexual orientation and race, ethnicity and racism.

Reporting on sex and gender	The human sera used in this studies were deidentified according to the IRB protocol from New York State Department of Health. Therefore, no sex and gender information has been linked to these serum samples.
Reporting on race, ethnicity, or other socially relevant groupings	The human sera used in this studies were deidentified according to the IRB protocol from New York State Department of Health. Therefore, no information of race, ethnicity, or other socially relevant groupings have been linked to these serum samples.
Population characteristics	The human sera used in this studies were deidentified according to the IRB protocol from New York State Department of Health. Therefore, no information of population characteristics has been linked to these serum samples.
Recruitment	We did not recruit patients for the serum collection. These serum samples were secondary use from the serodiagnostic laboratory in New York State Department of Health.
Ethics oversight	This study also involves secondary use of deidentified archival patient sera collected in previous studies and was approved by the Institutional Review Board (IRB) of New York State Department of Health and Baylor College of Medicine under protocol 565944-1 and H-46178, respectively. Analysis of deidentified patient data was carried out under a waiver of consent.

Note that full information on the approval of the study protocol must also be provided in the manuscript.

Field-specific reporting

Please select the one below that is the best fit for your research. If you are not sure, read the appropriate sections before making your selection.

[x] Life sciences [ ] Behavioural & social sciences [ ] Ecological, evolutionary & environmental sciences

For a reference copy of the document with all sections, see nature.com/documents/nr-reporting-summary-flat.pdf

Life sciences study design

All studies must disclose on these points even when the disclosure is negative.

Sample size	Assuming the groups compared with have the values greater than 1.69-fold differences, at least five replicates per group were utilized because attaining statistical significance ($p < 0.05$) between the negative control group and each of the other groups with 95% probability requires at least 5 replicates based on the power analysis.
Data exclusions	There are no data excluded in this study.
Replication	All biological replicates have been shown in this study.
Randomization	When performed the work invovled in in vivo studies using mice and human samples, samples were deidentified and randomized. The investigators are blinded for the group allocation.
Blinding	The samples involved in using mice and human samples were blinded during the analysis by the researchers.

Reporting for specific materials, systems and methods

We require information from authors about some types of materials, experimental systems and methods used in many studies. Here, indicate whether each material, system or method listed is relevant to your study. If you are not sure if a list item applies to your research, read the appropriate section before selecting a response.

Materials & experimental systems

n/a	Involved in the study	
[ ]	[x]	Antibodies
[x]	[ ]	Eukaryotic cell lines
[x]	[ ]	Palaeontology and archaeology
[ ]	[x]	Animals and other organisms
[x]	[ ]	Clinical data
[x]	[ ]	Dual use research of concern
[x]	[ ]	Plants

Methods

n/a	Involved in the study	
[x]	[ ]	ChIP-seq
[x]	[ ]	Flow cytometry
[x]	[ ]	MRI-based neuroimaging

Antibodies

Antibodies used	The goat anti-mouse IgG-HRP was ordered from Bethyl Lab/Fortis Life Science (#A90-116A). The goat anti-human IgG-HRP was ordered from Bethyl lab/Fortis Life Science (# A80-104). , Human IgG isotype control was order from Sigma-Aldrich, St. Louis, MO, catalog number AG100. Sheep anti-FH and Donkey anti Sheep IgG were ordered from Thermofisher with catalog number as SHAHU-FH and A16041, respectively. 1139c and 1193c were generated by us and described in the materials and methods or this manuscript.
Validation	The website for the validation of the commercially available antibodies are described below: oat anti-mouse IgG-HRP (https://www.fortislife.com/products/secondary-antibodies/goat-anti-mouse-igg-heavy-and-light-chain-antibody/BETHYL-A90-116), goat anti-human IgG-HRP (https://www.fortislife.com/products/secondary-antibodies/goat-anti-human-igg-fc-fragment-antibody/BETHYL-A80-104), Sheep anti-FH (https://www.thermofisher.com/antibody/product/Factor-H-Antibody-Polyclonal/SHAHU-FH) and Donkey anti Sheep IgG (https://www.thermofisher.com/antibody/product/Donkey-anti-Sheep-IgG-H-L-Secondary-Antibody-Polyclonal/A16041), the validation of 1139c and 1193c are in Supplemental Fig. 5

Animals and other research organisms

Policy information about studies involving animals; ARRIVE guidelines recommended for reporting animal research, and Sex and Gender in Research

Laboratory animals	Mus musculus mice
Wild animals	Not applicable
Reporting on sex	Only the female mice were included in this study.
Field-collected samples	Not applicable
Ethics oversight	All mouse experiments were performed in strict accordance with all provisions of the Animal Welfare Act, the Guide for the Care and Use of Laboratory Animals, and the PHS Policy on Humane Care and Use of Laboratory Animals. The protocol (Docket Number 22-451) was approved by the Institutional Animal Care and Use Agency of Wadsworth Center, New York State Department of Health.

Note that full information on the approval of the study protocol must also be provided in the manuscript.

Plants

Seed stocks	*Report on the source of all seed stocks or other plant material used. If applicable, state the seed stock centre and catalogue number. If plant specimens were collected from the field, describe the collection location, date and sampling procedures.*
Novel plant genotypes	*Describe the methods by which all novel plant genotypes were produced. This includes those generated by transgenic approaches, gene editing, chemical/radiation-based mutagenesis and hybridization. For transgenic lines, describe the transformation method, the number of independent lines analyzed and the generation upon which experiments were performed. For gene-edited lines, describe the editor used, the endogenous sequence targeted for editing, the targeting guide RNA sequence (if applicable) and how the editor was applied.*
Authentication	*Describe any authentication procedures for each seed stock used or novel genotype generated. Describe any experiments used to assess the effect of a mutation and, where applicable, how potential secondary effects (e.g. second site T-DNA insertions, mosiacism, off-target gene editing) were examined.*
